# Correlation between frontal QRS‐T angle, Tp‐e interval, and Tp‐e/QT ratio to coronary artery severity assessed with SYNTAX score in stable coronary artery disease patients

**DOI:** 10.1002/joa3.12756

**Published:** 2022-07-23

**Authors:** Fatma Özpamuk Karadeniz, Emine Altuntaş

**Affiliations:** ^1^ Department of Cardiology Private Metropolitan Hospital Konya Turkey; ^2^ Department of Cardiology Sancaktepe İlhan Varank Training and Research Hospital İstanbul Turkey

**Keywords:** electrocardiography, frontal QRS‐T angle, syntax score, Tp‐e interval, Tp‐e/QT ratio

## Abstract

**Background:**

It is known that a wide frontal QRS‐T(f[QRS‐T]) angle in the electrocardiography (ECG) is associated with poor cardiovascular outcomes. The Tp‐e (the interval from the peak to the end of the T wave) interval and Tp‐e/QTc ratio show the dispersion of repolarization, and increased levels lead to ventricular arrhythmogenesis in congenital channelopathies and coronary heart disease. In this study, we aimed to investigate the relationship between f(QRS‐T), Tp‐e interval, and Tp‐e/QTc ratio and SYNTAX score in stable coronary artery disease (SCAD) patients.

**Methods:**

A total of 403 patients who performed coronary angiography for SCAD were included. The study population was divided into two groups based on the SYNTAX score. Group 1 included 248 patients (high SYNTAX score > 0), and group 2 included 155 patients (low SYNTAX score = 0). SYNTAX score was calculated using an online SYNTAX score calculator from the coronary angiography images of each patient. The f(QRS‐T) angle (QRS angle minus T angle) was calculated from the automated reports of the 12‐lead ECG device. Tp‐e interval and Tp‐e/QTc ratio and other electrocardiographic parameters were recorded.

**Results:**

The mean SYNTAX score in group 1 was 8. F(QRS‐T) angle, Tp‐e duration, Tp‐e/QT, and Tp‐e/QTc were significantly higher in group 1 compared with group 2. In the multivariate regression analysis, F(QRS‐T) angle and Tp‐e duration were independent predictors for SYNTAX scores in SCAD patients.

**Conclusions:**

Our study showed that Tp‐e interval, Tp‐e/QTc ratio, and f(QRS‐T) angle were increased in patients with higher SYNTAX scores in patients with SCAD patients.

## Graphical abstract

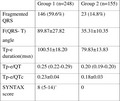

## 1 INTRODUCTION

Coronary artery disease is the leading cause of death all over the world and continues to be major pathology associated with sudden cardiac death.^1^ In ischemia, due to obstructive coronary atherosclerosis, electrical heterogeneity occurs in the vulnerable ventricular myocardium and ventricular arrhythmias occur due to repolarization abnormalities.^2^ These abnormalities reflect surface electrocardiography (ECG). ECG is a diagnostic tool that is frequently found and used in many clinics, where many parameters can be easily evaluated at the same time.

The Tpeak‐end (Tp‐e) interval refers to the time from the peak T to the end of the T wave. The Tp‐e interval is a repolarization parameter showing the global and transmural repolarization dispersion.^3^ Many studies have shown the relationship between the peak and end of the T wave (Tp‐e) and the Tp‐e/QTc ratio with malignant ventricular arrhythmias in electrocardiography.^4^ Correcting the Tp‐e for heart rate using either Bazett's (n/√RR) or Fridericia's (n/_3_√RR) formula improved the predictive value for sudden cardiac death.^5^

The spatial QRS‐T angle is a new marker of ventricular repolarization and it is defined the spatial angle as the difference in angle between the direction of ventricular depolarization (QRS) and the direction of ventricular repolarization (T wave).^6^ Calculating the spatial QRS‐T angle is complex and requires a software program and takes a long time. Instead, the frontal QRS‐T(f(QRS‐T)) angle, which is calculated automatically in ECG devices, has been developed and has been shown to be parallel to the spatial angle. In recent years, the widened f(QRS‐T) angle was found predictor of all‐cause mortality and cardiac death.^7^

The SYNTAX score shows the complexity of coronary artery disease and it is recommended by the current guidelines in making the decision of percutaneous coronary intervention or coronary artery bypass grafting.^8^, ^9^ There are few studies have examined the association between the coronary artery severity with the f(QRS‐T) angle, Tp‐e interval, and Tp‐e/QT ratios in SCAD patients. In the study, the f(QRS‐T) angle predicted SYNTAX scores in NSTEMI patients.^10^ According to our hypothesis, as the amount and extent of ischemia increases, the SYNTAX score also increases and this reflects in the surface ECG. In this study, we aimed to evaluate the relationship between the f(QRS‐T) angle, Tp‐e interval, and Tp‐e/QT ratios evaluated from the surface ECG, and the complexity of coronary artery disease, which was evaluated with the SYNTAX score in patients who underwent coronary angiography for SCAD.

## 2 MATERIALS AND METHODS

### 2.1 Study population

Our study was performed retrospectively and cross‐sectionally in patients diagnosed with stable angina pectoris who presented to our hospital between January 2019 and March 2022 in the cardiology clinic. The study was conducted at two centers. Four hundred and three patients who underwent coronary angiography for stable angina pectoris were enrolled in the study. Of the 539 patients with SCAD who were screened retrospectively, 136 were excluded because they did not meet the inclusion criteria or had exclusion criteria. Patients with any of the following were excluded: previous coronary artery bypass grafting, complete or incomplete bundle branch block, atrial fibrillation, paced rhythm, and significant valvular heart disease. Baseline demographic and clinical characteristics were reviewed. The study complied with the principles outlined in the Declaration of Helsinki and was approved by a local ethics committee.

### 2.2 Electrocardiography

**FIGURE 1:**
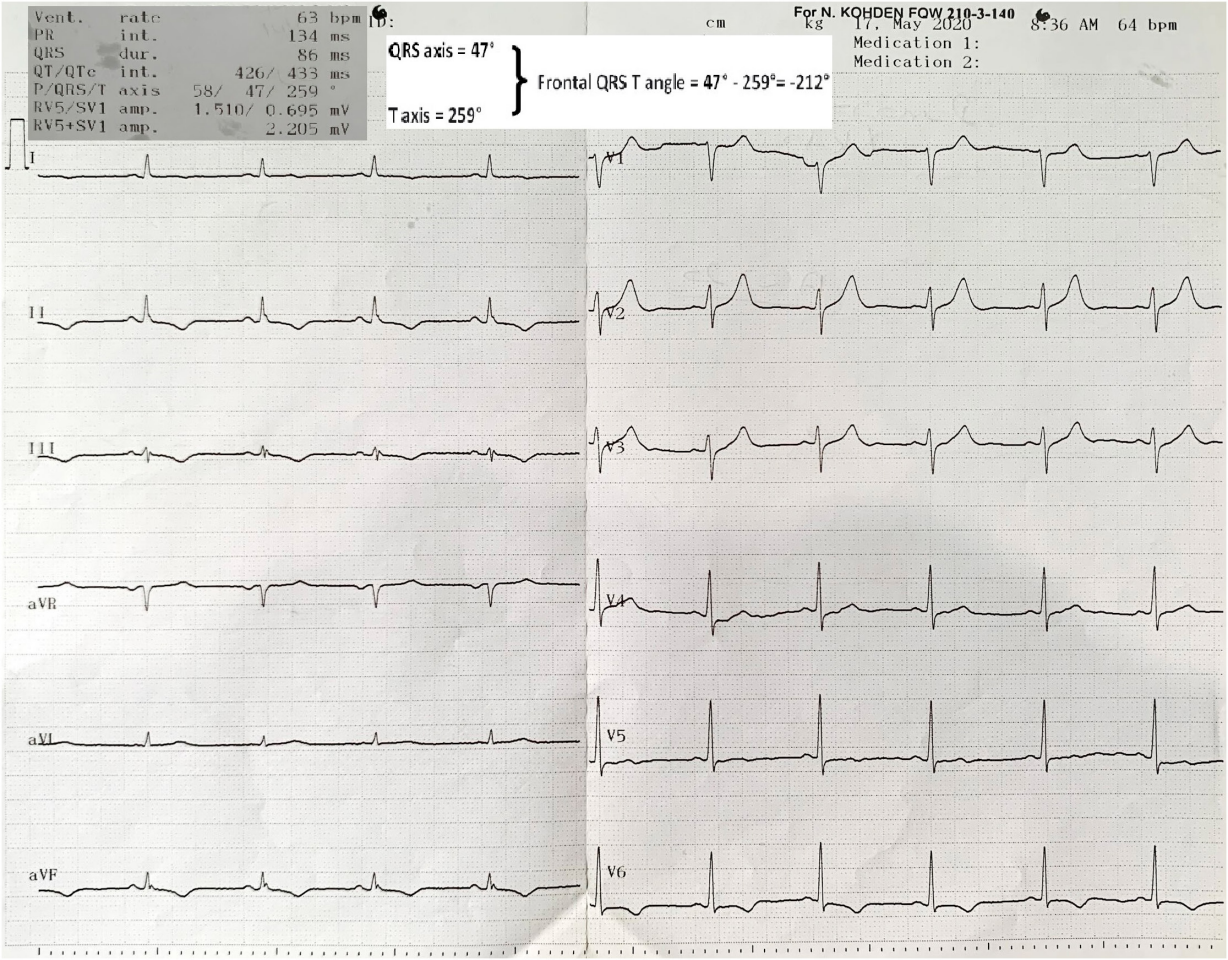
Measurement of frontal QRS‐T angle from the automatic report of surface ECG

The 12‐lead ECG (Nihon Kohden Corporation, Cardiofax M Model ECG‐1250, Tokyo, Japan) was recorded at a paper speed of 25 mm/s in the supine position after resting for at least 10 minutes. To decrease the error measurements, parameters were obtained by using the software after x400% magnification. ECG measurements of QT and Tp‐e intervals were performed by two cardiologists who were blinded to the patient data. Subjects with U waves on their ECGs were excluded from the study. An average value of three readings was calculated for each lead. The QT interval was measured from the beginning of the QRS complex to the end of the T wave and corrected for heart rate using the Bazett formula: cQT = QT√ (RR interval). The Tp‐e interval was defined as the interval from the peak of the T wave to the end of the T wave. Measurements of the Tp‐e interval were performed from V5 precordial lead. The Tp‐e/QT ratio was calculated from these measurements. Frontal QRS‐T angle is obtained from the automatic report part of surface ECG. It is equal to the difference between the frontal QRS axis and the T axis (frontal QRS‐T angle = QRS axis – T axis) and it was categorized as normal (<100°) or abnormal (≥100°) values (Figure 1).

### 2.3 Laboratory and angiographic analyses

Demographic characteristics of the patients, fasting blood glucose, creatinine, low‐density lipoprotein cholesterol (LDL‐C), triglyceride, hemoglobin, neutrophil, lymphocyte and platelets, C‐reactive protein, and ECG, echocardiography, and coronary angiography images were examined from the hospital records.

Hypertension was defined as the documentation of systolic blood pressure of 140 mm Hg and/or diastolic blood pressure of ≥90 mm Hg in at least two measurements or as the active use of any antihypertensive agent. Diabetes was defined as the fasting plasma glucose level of >126 mg/dl or the glucose level of >200 mg/dl in any measurement or actively using an antidiabetic agent. Coronary heart disease was diagnosed by a history of myocardial infarction and revascularization, by angiographic >50% stenosis of at least one major coronary artery. Dyslipidemia mellitus was defined as the fasting total cholesterol level of >200 mg/dl or actively using an antilipidemic agent.

The patients were divided into two groups on the basis of SYNTAX scores as low (=0) and moderate–high risk (>0). Images of the angiogram were evaluated by two experienced interventional cardiologists. The SYNTAX 1 score was calculated using the site http://www.syntaxscore.com. Interobserver and intraobserver coefficients of variation were 2.2% and 2.4%, respectively.

### 2.4 Statistical analysis

IBM SPSS Statistics for Windows, Version 18.0 (IBM Corp., Armonk, NY, USA) was used to perform the statistical analysis. Kruskal–Wallis test was used to assess the normality of the distribution of the variables. Quantitative variables with a normal distribution were specified as the mean ± standard deviation and nonnormally distributed variables were specified as median (25%–75% range). Categorical variables were shown as numbers and percentage values. The nonnormally distributed variables were assessed with the Mann–Whitney *U* test, whereas normally distributed variables were assessed with an independent sample Student's *t* test. The categorical variables were analyzed with a Chi‐squared test. Univariate and multivariate regression analyses identified factors related to clinical endpoints. *p* value of <0.05 was accepted as statistically significant.

## 3 RESULTS

**FIGURE 2:**
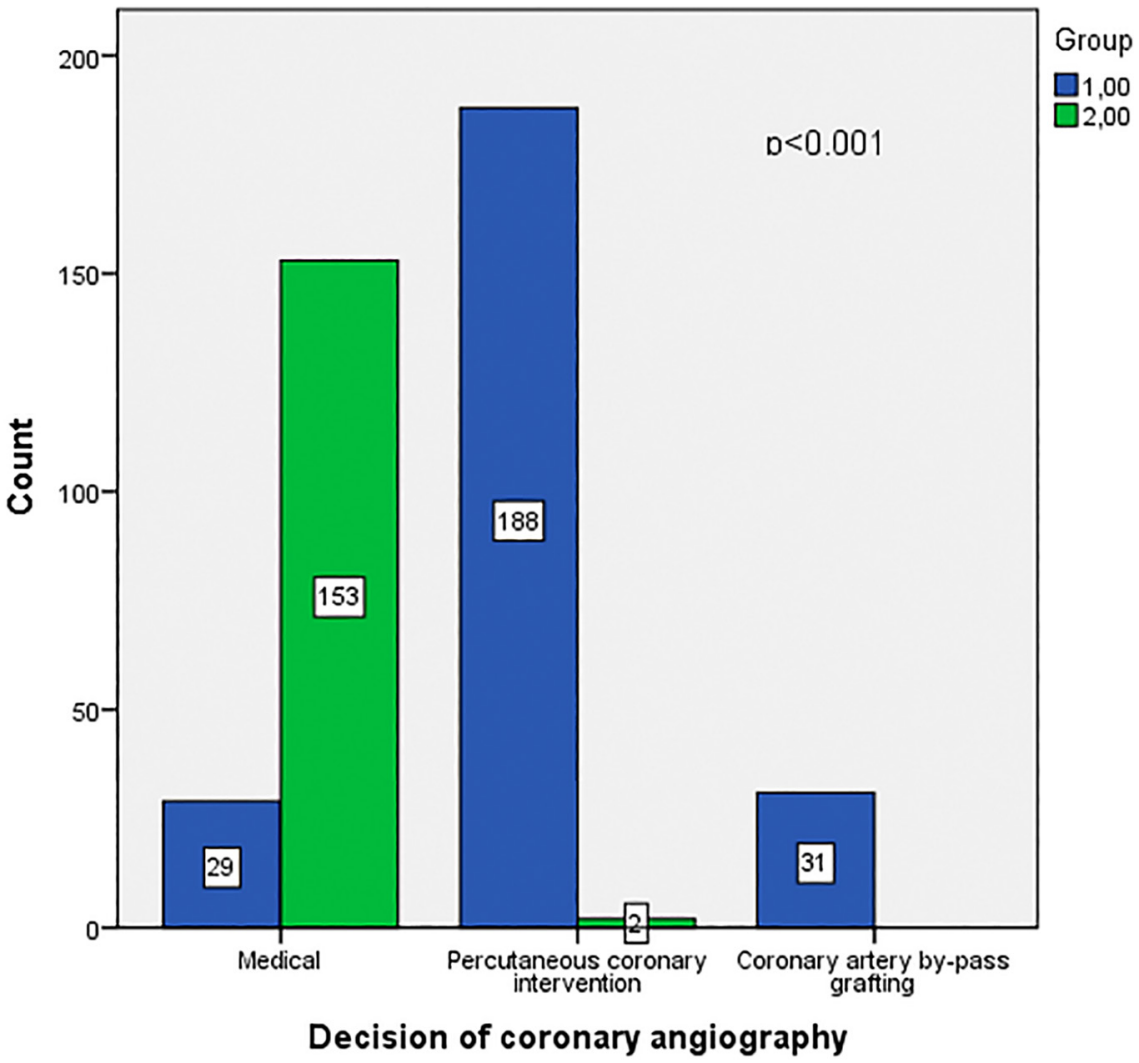
Decision of coronary artery angiography result

A total of 403 patients were categorized into two groups according to SYNTAX scores. Group 1 included 248 SCAD patients with the SYNTAX score of >0 and group 2 included 155 patients with the SYNTAX score of 0. The mean SYNTAX score in group 1 was 8. Of 403 patients, 229 (56.8%) were males. The mean age of the patients was 63.74 ± 11.61. Baseline demographic characteristics, laboratory, and transthoracic echocardiographic findings of the study population are presented in Tables 1 and 2. There was no difference between the two groups regarding age, gender, diabetes mellitus (DM), hypertension (HT), chronic renal failure, history of coronary artery disease, and dyslipidemia. Also, ACEI or ARB, beta‐blocker, calcium channel blocker, and statin use were similar in both groups (*p* > .05). According to the coronary angiography results, 188 patients underwent PCI in group 1, whereas 2 patients underwent PCI in group 2. In groups 1 and 2, 29 patients and 188 patients were treated medically, respectively. Coronary artery bypass grafting decision was given to the 31 patients in group 1. The decisions of coronary angiography are summarized in Figure 2.

**TABLE 1:** Demographic and clinical characteristics of the groups

	Total	Group 1 (*n* = 248)	Group 2 (*n* = 155)	*p*
Male	229 (56.8%)	155 (62.5%)	74 (47.7%)	.003^a^
Age	66.60 ± 12.36	67.44 ± 12.18	65.26 ± 12.60	.088^b^
Smoking	80 (19.9%)	53 (21.4%)	27 (17.4%)	.201^a^
History of CAD	141 (35%)	80 (32.3%)	61 (39.4%)	.089^a^
HT	299 (74.2%)	178 (71.8%)	121 (78.1%)	.098^a^
HL	199 (49.4%)	122 (49.2%)	77 (49.7%)	.503^a^
DM	156 (38.7%)	89 (35.9%)	67 (43.2%)	.086^a^
CRF	1 (0.2%)	1 (0.4%)	0	.615^a^
Beta blocker	143 (35.5%)	91 (36.7%)	52 (33.5%)	.297^a^
Calcium channel blocker	70 (17.4%)	47 (19%)	23 (14.8%)	.178^a^
ACEI or ARB	195 (48.4%)	121 (48.8%)	74 (47.7%)	.459^b^
Statin	156 (38.7%)	95 (38.3%)	61 (39.4%)	.457^b^

*Note*: Abbreviations: ACEI, angiotensin‐converting enzyme inhibitors; ARB, angiotensin receptor blockers; CAD, coronary artery disease; CRF, chronic renal failure; DM, diabetes mellitus; HL, hyperlipidemia; HT, hypertension.

^a^ Fisher's exact test.

^b^ Independent sample *t* test.

**TABLE 2:** Laboratory tests, transthoracic echocardiography results, and electrocardiographic results of the groups

	Group 1 (*n* = 248)	Group 2 (*n* = 155)	*p*
Glucose (mg/dl)	123 (102–159)¨	106 (96–138.50)¨	<.001^b^
Total cholesterol (mg/dl)	196.07 ± 48.21	216.28 ± 37.85	.126^a^
Triglyceride(mg/dl)	163 ± 28.2	141.9 ± 27.6	.129
LDL (mg/dl)	124.59 ± 40.01	123.73 ± 35.31	.827^a^
Hba1c (%)	5.6 ± 0.92	5.0 ± 0.42	.76
Serum creatinine (mg/dl)	0.99 (0.79–1.6)¨	0.85 (0.73–1.18)¨	<.001^b^
Ejection fraction (%)	52.92 ± 10.53	57.71 ± 7.99	<.001^a^
Heart rate (sec)	77.57 ± 14.29	75.58 ± 13.30	.155^a^
Fragmented QRS	146 (59.6%)	23 (14.8%)	<.001^a^
F(QRS‐T) angle	89.87 ± 27.82	35.31 ± 10.35	<.001^a^
Tp‐e duration (msn)	100.51 ± 18.20	79.83 ± 13.83	<.001^a^
QT duration (msn)	386.11 ± 36.31	383.87 ± 31.94	.528
QTc duration (msn)	424.35 ± 32.06	421.41 ± 28.44	.351^a^
Tp‐e/QT	0.25 (0.22–0.29)¨	0.20 (0.19–0.20)¨	<.001^b^
Tp‐e/QTc	0.23 ± 0.04	0.18 ± 0.03	<.001^a^
SYNTAX score	8 (5–14)¨	0	<.001^b^

*Note*: Abbreviations: dL, deciliter; LDL, low‐density lipoprotein; mg, milligram; msn, millisecond; QTc, corrected QT duration; sec, second; Tp‐e, Tpeak‐end.

¨25%–75% quartile.

^a^ Independent sample *t* test.

^b^ Mann–Whitney *U* test.

Left ventricle ejection fraction (LVEF) (*p* = <.001) was lower in group 1. Glucose and serum creatinine were significantly higher in group 1 compared with group 2 (p = <.001, <.001) (Table 2). QRS‐T angle, Tp‐e duration, Tp‐e/QT, Tp‐e/QTc, and SYNTAX scores were higher in group 1 compared with group 2 (*p* = <.001, <.001, <.001, <.001, and <.001) (2). Except for these, ECG parameters fragmented QRS frequency was higher in group 1.

Univariate regression analysis was performed in one model. The QRS‐T angle, Tp‐e duration, total cholesterol, and LDL were detected as independent predictors in group 1. A positive relationship was found between the SYNTAX score and these variables. According to multivariate regression analysis, QRS‐T angle, Tp‐e, and EF remained independent predictors of higher SYNTAX scores in group 1 (*p* = .001) (Table 3). While there is a positive relationship between QRS‐T angle, Tp‐e duration, and SYNTAX score, a negative relationship was found with EF.

**TABLE 3:** Univariate and multivariate regression analyses on the risk factors associated with SYNTAX score in group 1

	Univariate analysis		Multivariate analysis *p* = .001, *R* ^2^ = .078	
Variable	Β	*p*	Β	*p*
QRS‐T angle	0.019 (0.005 to 0.033)	.010	0.016 (0.001 to 0.030)	.016
Tp‐e duration	0.057 (0.005 to 0.108)	.030	0.055 (0.004 to 0.106	.034
QT duration	0.016 (−0.009 to 0.042)	.210		
QTc duration	0.021 (−0.008 to 0.050)	.162		
Tp‐e/QT	13.708 (−4.749 to 32.164)	.145		
Tp‐e/QTc	7.793 (−12.590 to 28.176)	.452		
Glucose	0.003 (−0.0127to 0.019)	.661		
Total cholesterol	0.051 (0.021 to 0.081)	.001		
LDL	0.037 (0.014 to 0.060)	.002	0.053 (−0.049 to 0.155)	.308
Ejection fraction	−0.007 (−0.108 to 0.094)	.886	−0.09 (−0.019 to −0.001)	.003

*Note*: Abbreviations: LDL, low‐density lipoprotein; QTc, corrected QT duration.

## 4 DISCUSSION

The results of our study demonstrated that f(QRS‐T) angle, Tp‐e interval, and Tp‐e/QTc ratios were considerably prolonged in obstructive SCAD patients with a high SYNTAX score. In the SYNTAX score, more than each coronary lesion with diameter stenosis ≥50% in vessels ≥ 1.5 mm must be scored. For this reason, in our study group 1 with a SYNTAX score of >0 represented obstructive SCAD patients, and group 2 with a 0 SYNTAX score represented nonobstructive SCAD patients. As the degree and extent of ischemia increase, more repolarization abnormalities occur on the surface ECG. As a result, it is expected that the changes in ECG will be more in patients with more critical and diffuse coronary artery stenosis calculated by the SYNTAX score. Since we found few studies in the literature on this subject, we planned to conduct a study on this subject in SCAD patients. Except for these electrocardiographic parameters, fragmented QRS frequency was found higher in group 1 with a high SYNTAX score.

The surface ECG is still the perfect tool to reflect the electrical activity of the heart. The alterations of QT, QTc, and Tp‐e (Tpeak‐Tend) intervals and the Tp‐e/QT and Tp‐e/QTc ratios all of which are parameters of ventricular repolarization.^3^ Several studies showed that increased electrocardiographic parameters are associated with lethal arrhythmic events and mortality in Brugada, long QT syndromes, and coronary artery disease patients.^3^, ^5^, ^11^, ^12^ In the review included 155 856 patients, the Tpeak‐end interval was found a useful risk stratification tool in different diseases and in the general population.^3^ The increased Tp‐e interval was found predictor of in‐hospital and long‐term mortality in STEMI and acute myocardial infarction patients.^13^, ^14^ Tp‐e interval, Tp‐e/QT ratio, and Tp‐e/QTc were found significantly increased in slow coronary flow patients.^15^ These electrocardiographic parameters were found to be associated with poor prognosis and mortality in many disease groups except cardiovascular disease.^16^, ^17^, ^18^, ^19^ Except for the association with arrhythmic events and mortality, in a study of 421 patients with acute coronary syndrome, increased Tp‐e interval and Tp‐e/QT ratio rates were found to be correlated with higher SYNTAX and GRACE scores.^20^ There was found association between the prolonged Tp‐e interval and increased Tp‐e/QT and Tp‐e/QTc ratios with the presence of CAD, especially in patients with the acute ischemic syndromes.^21^ In the presented study, there was found significant association between coronary artery complexity assessed with SYNTAX scores and electrocardiographic parameters including Tp‐e, Tp‐e/QT, and Tp‐e/QTc.

Frontal QRS‐T angle is another parameter to assess ventricular repolarization. The widening of the QRS‐T angle, which is the difference between the mean QRS angle and the mean T angle, has been shown to be a predictor of cardiac mortality.^22^ The QRS‐T angle, whether it is measured by spatial or frontal methods, varies by gender and age. Generally, women have a smaller angle at baseline than men, and in both sexes, the angle widens with age. Normal frontal angles are generally smaller than normal spatial angles.^22^ In a meta‐analysis, involving 164,171 patients, both frontal and spatial QRS‐T angles were found to be predictive of all‐cause and cardiac death.^7^ An increased frontal QRS‐T angle of >90° was found significant predictor of a composite end point of death, appropriate implantable cardioverter‐defibrillator shock, or resuscitated cardiac arrest in nonpaced, mild to moderately symptomatic patients with nonischemic cardiomyopathy.^23^ Aro et al. revealed that the frontal QRS‐T angle of ≥100° increased the risk of arrhythmic death as a result of an altered T‐wave axis in a middle‐aged general population.^24^ In the study performed on 269 patients with NSTEMI, the f(QRS‐T) angle predicted the SYNTAX score.^6^ Our study supported the study that the f(QRS‐T) angle predicted SYNTAX scores in SCAD patients. To the best of our knowledge, there is no study that compares the f(QRS‐T) angle and SYNTAX association in SCAD patients. According to the present study result, before coronary angiography in SCAD patients, a preliminary clinic idea about the extent and complexity of coronary artery disease can be obtained by looking at the f(QRS‐T) angle. Most of the published studies revealed an association between these electrocardiographic parameters showing repolarization abnormalities and cardiac mortality, but this study differs in showing the extent of coronary artery disease. If long‐term follow‐up of the patients had been done, the arrhythmic event rates could have been found to be high.

## 5 LIMITATIONS

The primary limitation of our study is the relatively small sample population, and the follow‐up of patients could not be done. An association between ventricular arrhythmias with f(QRS‐T), Tp‐e interval, and Tp‐e/QT ratio was not achieved.

## 6 CONCLUSION

Our study strongly supported that increased Tp‐e, Tp‐e/QT, Tp‐e/QTc ratios, and widened f(QRS‐T) angle were associated with higher SYNTAX scores in SCAD patients. Adding frontal QRS angle, Tp‐e intervals, and Tp‐e/QTc ratios can be used to predict the severity and complexity of coronary artery disease before diagnostic coronary angiography in SCAD patients.

FUNDING INFORMATION

No funding was received to assist with the preparation of this manuscript. The authors have no relevant financial or nonfinancial interests to disclose. The study was approved by a local ethic committee (Professor Dr. İlhan Varank Training and Research Hospital ethics committee). The study is a retrospective cross‐sectional study. For this reason, the patient consent statement was not taken. The clinical trial number is absent.
